# Palliative high tibial osteotomy achieves comparable outcomes to corrective osteotomy in varus knees with medial compartment osteoarthritis: A long‐term clinical and radiological retrospective study

**DOI:** 10.1002/jeo2.70610

**Published:** 2026-02-26

**Authors:** Alessio Maione, Giuseppe Fedele, Pierrenzo Pozzi, Matteo Davide Parmigiani, Alessandra Menon, Filippo Calanna, Riccardo Compagnoni, Paolo Ferrua, Massimo Berruto, Pietro Simone Randelli

**Affiliations:** ^1^ U.O.C. I Clinica Ortopedica ASST Gaetano Pini—CTO Milan Italy; ^2^ Università degli Studi di Milano Milan Italy; ^3^ Department of Biomedical Sciences for Health Università degli Studi di Milano Milan Italy; ^4^ Department of Biomedical, Surgical and Dental Sciences Università degli Studi di Milano Milan Italy

**Keywords:** corrective osteotomy, high tibial osteotomy, joint line obliquity, knee, lower limb alignment, palliative osteotomy, varus knee

## Abstract

**Purpose:**

The long‐term outcomes of palliative high tibial osteotomy (PO) remain insufficiently defined. This study compared the clinical and radiological outcomes of PO with those of corrective osteotomy (CO) in patients with varus knee deformity. Secondary aim was to evaluate arthroplasty‐free survival. It was hypothesized that PO and CO would yield comparable results and similar conversion rates to total knee arthroplasty (TKA).

**Methods:**

A retrospective cohort study was conducted on patients who underwent lateral closing wedge‐high tibial osteotomy (LCW‐HTO) between 2001 and 2017. Patients with extra‐articular varus deformity (hip–knee–ankle angle [HKA] < 177°, lateral distal femoral angle [LDFA] > 90° or medial proximal tibial angle [MPTA] < 85°) were assigned to the CO group. Those with intra‐articular deformity and normal MPTA and LDFA were assigned to the PO group. Radiographic evaluation included HKA, MPTA, LDFA, joint line obliquity (JLO) and joint line convergence angle (JLCA). Clinical outcomes were assessed using the Hospital for Special Surgery (HSS) score, International Knee Documentation Committee (IKDC) subjective score, Numeric Rating Scale (NRS) for pain, Tegner Activity Scale and Crosby–Insall grading.

**Results:**

Forty patients were included, 20 in each group. The mean age was 49 ± 11 years, and the mean follow‐up was 10.5 ± 2.9 years. Preoperative HKA averaged 174° ± 3.3° and improved to 179° ± 1.5°. Moreover, 25% of PO patients and 10% of CO patients postoperatively exceeded the JLO threshold of ≤4°. Both groups demonstrated significant clinical improvement, with no between‐group differences except for higher HSS scores in the PO group. Osteoarthritis (OA) progression was limited, and TKA conversions were infrequent.

**Conclusion:**

PO may represent an effective joint‐preserving option for intra‐articular varus deformity. Despite less optimal correction of JLO and JLCA, PO achieved clinical and radiological outcomes comparable to CO, with similarly low conversion rates to TKA.

**Level of Evidence:**

Level IV, retrospective cohort study.

**Abbreviations**
ACLR: anterior cruciate ligament reconstruction
CO: corrective osteotomy
HKA: hip–knee–ankle axis
HSS: Hospital for Special Surgery
ICC: intraclass correlation coefficient
IKDC: International Knee Documentation Committee
JLCA: joint line congruency angle
JLO: joint line obliquity
KL: Kellgren–Lawrence
LCW‐HTO: lateral closing wedge‐high tibial osteotomy
LDFA: lateral distal femoral angle
MPTA: medial proximal tibial angle
NRS: Numeric Rating Scale
OA: osteoarthritis
PO: palliative osteotomy
TBVA: tibial bone varus angle
TKA: total knee arthroplasty
UKA: unicompartmental knee arthroplasty

## INTRODUCTION

Joint‐preserving procedures such as high tibial osteotomy (HTO) may delay or avoid the need for prosthetic replacement in younger and more active patients with unicompartmental osteoarthritis (OA) [24]. HTO can be broadly classified into corrective osteotomy (CO) and palliative osteotomy (PO), depending on the underlying deformity. CO is indicated in cases of measurable extra‐articular malalignment, such as tibial varus deformity (medial proximal tibial angle [MPTA] < 85°) or femoral deformity (lateral distal femoral angle [LDFA] > 90°), and its goal is to restore the mechanical axis and normalize joint biomechanics [12, 27].

In contrast, PO is performed in younger, active patients who have failed conservative treatment but present with intra‐articular deformity, often secondary to conditions such as meniscectomy or meniscal extrusion, without a correctable bony deformity [3]. The choice between unicompartmental knee arthroplasty (UKA) and osteotomy depends on multiple factors, including age, activity level, lower limb alignment and knee stability [9].

PO primarily aims to relieve symptoms and improve functional outcomes, and it has demonstrated favourable clinical and radiological results at medium‐ to long‐term follow‐up [4]. One of its key advantages is the high survival rate, which allows patients to maintain mobility and quality of life for several years before requiring more invasive arthroplasty procedures [22, 28].

This study primarily aimed to evaluate the long‐term effectiveness of PO compared to CO performed in patients with localized medial OA and varus knee deformity. The primary objective was to compare PO and CO groups in clinical and radiological outcomes, while the secondary objective was to assess arthroplasty‐free survival. The primary hypothesis was that PO and CO yielded comparable improvements in clinical and radiological scores. The secondary hypothesis was that the two procedures resulted in similar conversion rates to arthroplasty.

## MATERIALS AND METHODS

The study was designed based on the criteria of the Declaration of Helsinki and approved by the local ethics committee (Comitato Etico Milano AREA 2—protocol number IOGPMB02). Patients were recruited on voluntary basis, and all of them signed informed consent.

A retrospective analysis was conducted on all the patients with malalignment of the lower limbs treated with a lateral closing wedge‐high tibial osteotomy (LCW‐HTO). All patients had previously undergone conservative treatment, including physical therapy and intra‐articular injections, without achieving sustained clinical benefit. All the surgeries were performed by a senior knee surgeon between 2001 and 2017.

The following inclusion criteria were adopted: the presence of symptomatic medial overload or OA (Kellgren–Lawrence [KL] Grades I–III) [18] with varus alignment, both intra‐articular and extra‐articular (hip–knee–ankle angle [HKA] > 170°), pre‐ and postoperative radiographs (anteroposterior weight‐bearing long‐leg, standard weight‐bearing knee views, Rosenberg views), age > 18 years at the time of surgery and a minimum clinical follow‐up of 5 years after surgery. Patients were excluded from participating in the study in cases of previous osteotomies on the same limb, previous fractures of the same limb, ipsilateral hip prosthesis and extension of OA KL Grade > II to the lateral or patellofemoral compartments as a contraindication to the osteotomy procedure. Patients with an HKA < 165° were excluded from the study because either a double‐level or a two‐stage osteotomy or an external fixation system was indicated to avoid an excessive joint line inclination and to reduce the risk of intraoperative fractures or hypo‐corrections of the deformity.

Preoperatively, all osteotomies were planned as described by Miniaci [1], aiming to shift the mechanical axis so that it falls through the centre or just medial to the centre of the knee.

Standing long‐leg and knee radiographs were collected for each patient before surgery and at last follow‐up to assess the grade of knee OA, the lower limb alignment with the HKA, the MPTA, the LDFA, the joint line obliquity (JLO) angle and the joint line congruency angle (JLCA). Patients were recontacted and called in to undergo radiographs.

Patients were divided into CO and PO groups according to radiographic parameters. Extra‐articular deformities (MPTA < 85° and/or LDFA > 90°) were classified as CO, whereas intra‐articular deformities with normal MPTA and LDFA but increased JLCA were classified as PO. The HKA angle was used only to confirm varus alignment (HKA < 177°) and not as a primary criterion for grouping.

The clinical outcome was evaluated at the baseline and at the last follow‐up with a clinical evaluation and through several validated scores: Hospital for Special Surgery (HSS) knee score [11], International Knee Documentation Committee (IKDC) Subjective Knee Form [14], Tegner Activity Scale [33] and the Numeric Rating Scale (NRS) for pain [11]. Clinically meaningful improvements were defined according to established minimal clinically important difference (MCID) thresholds: 9 points for the HSS knee score, 12 points for the IKDC subjective score, 0.5–1.0 levels for the Tegner Activity Scale and a reduction of ≥2 points or ≥30% for the NRS for pain [2, 8]. The overall patient satisfaction was collected according to the Insall and Crosby grading system (Crosby–Insall) at final follow‐up [5].

After surgery, all the patients began immediate passive and active joint mobilization. Partial weight‐bearing as tolerated was permitted, with the use of a knee brace locked in extension and walking aids for 4 weeks. At the same time, peri‐ or postoperative complications and failures were registered throughout the study period. After 4 weeks, the patients discontinued the use of the brace and gradually discontinued the use of crutches. A physiokinesitherapy protocol was implemented to restore muscle strength and trophism.

### Radiographical measurements

**Figure 1:**
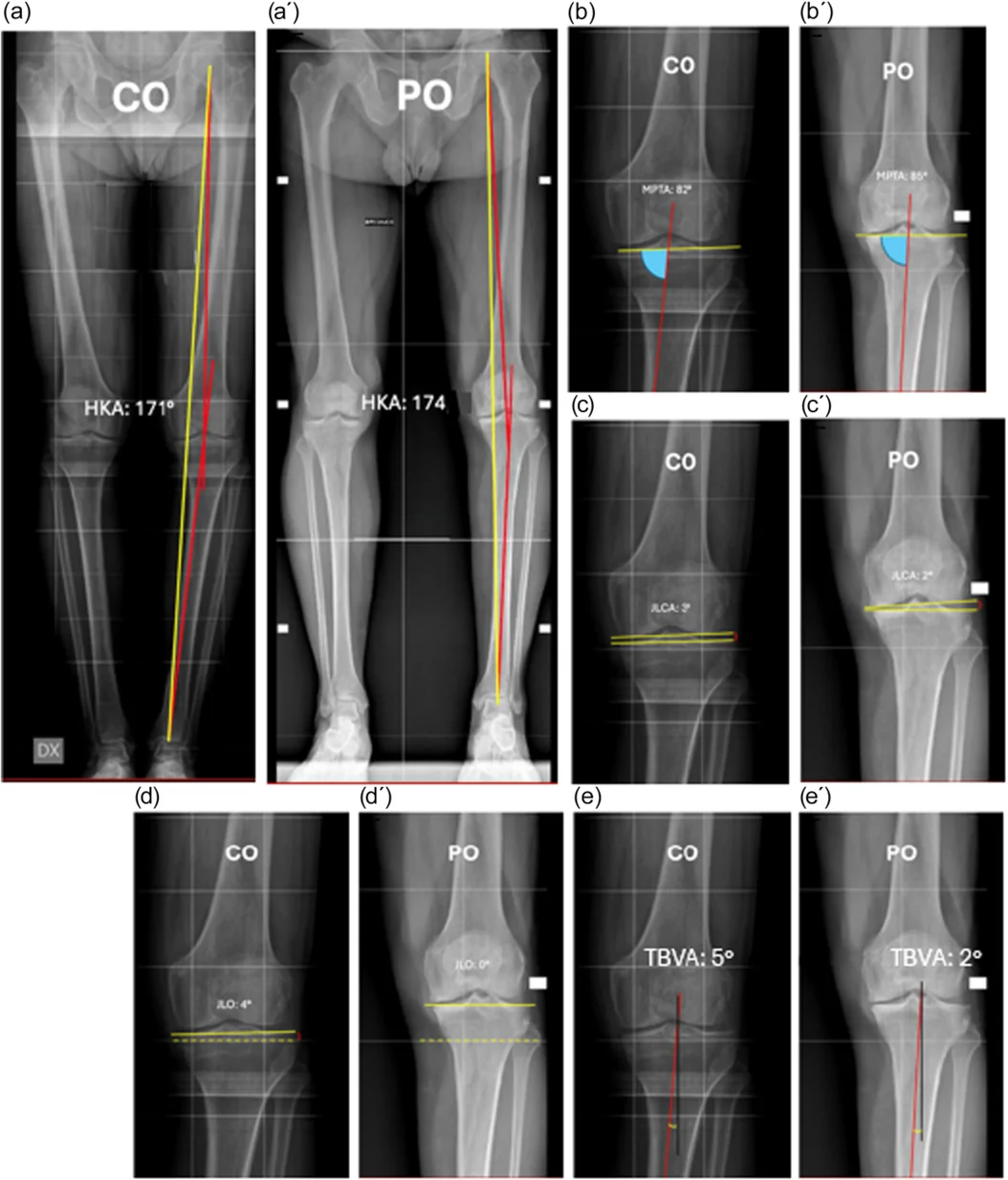
Preoperative radiographical measurements. (a) and (a′) Hip–knee–ankle angle (HKA) for corrective osteotomy (CO) and palliative osteotomy (PO) group (in red the mechanical axes, in yellow the Mikulicz line). (b) and (b′) Mechanical medial proximal tibial angle (MPTA) for CO and PO groups. (c) and (c′) Joint line convergency angle (JLCA) for CO and PO group. (d) and (d′) Joint line obliquity (JLO) for CO and PO groups (in solid line the tangent to the tibial plateau and in dashed line the reference to the ground). (e) and (e′) Tibial bone varus angle (TBVA) for CO and PO groups with the mechanical axis of the tibia (in black) and the proximal tibia epiphyseal axis (in red).

**Figure 2:**
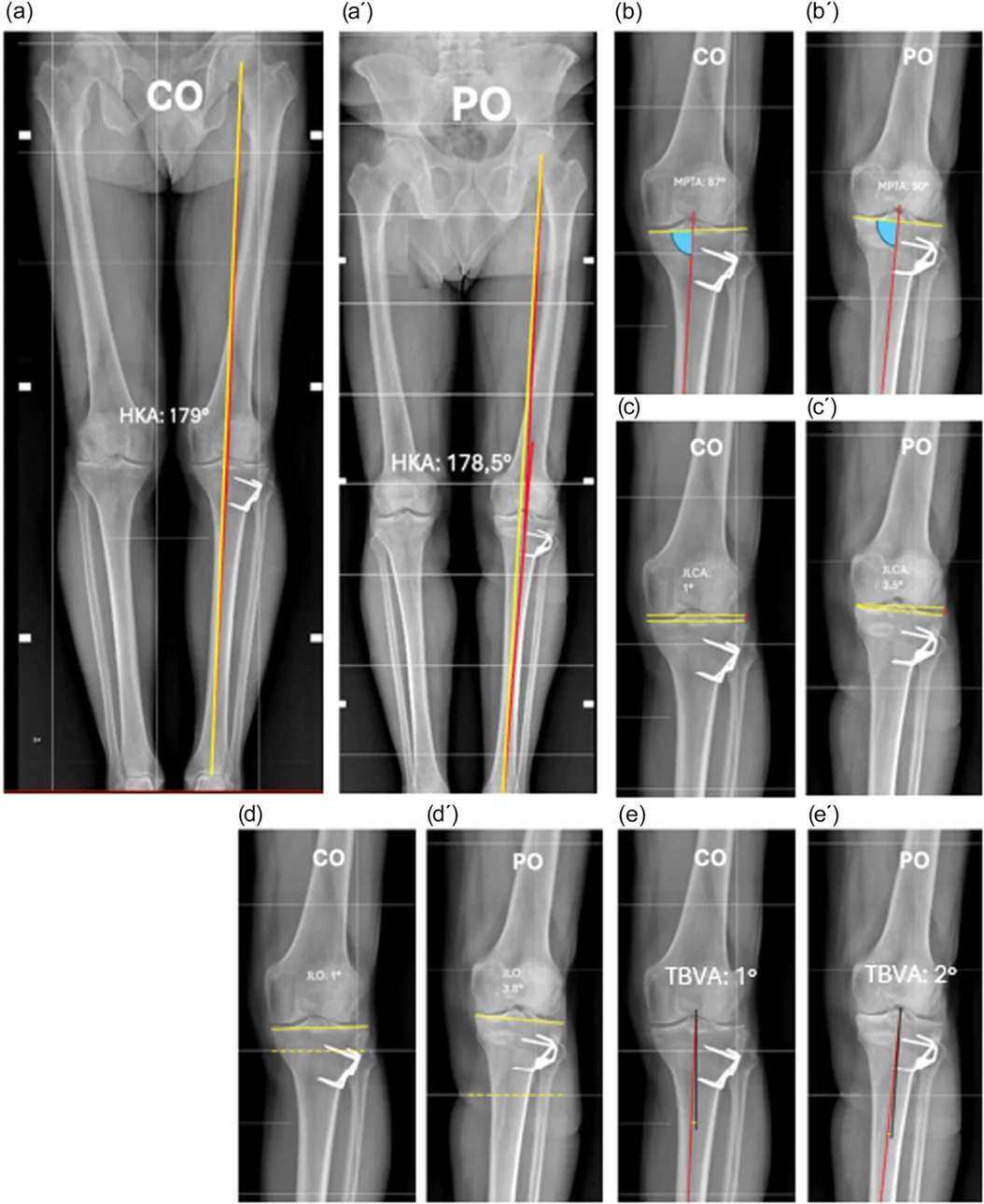
Preoperative radiographical measurements. (a) and (a′) Hip–knee–ankle angle (HKA) for corrective osteotomy (CO) and palliative osteotomy (PO) group (in red the mechanical axes, in yellow the Mikulicz line). (b) and (b′) Mechanical medial proximal tibial angle (MPTA) for CO and PO groups. (c) and (c′) Joint line convergency angle (JLCA) for CO and PO groups. (d) and (d′) Joint line obliquity (JLO) for CO and PO group (in solid line the tangent to the tibial plateau and in dashed line the reference to the ground). (e) and (e′) Tibial bone varus angle (TBVA) for CO and PO groups with the mechanical axis of the tibia (in black) and the proximal tibia epiphyseal axis (in red).

Long‐leg anteroposterior weight‐bearing radiographs and weight‐bearing knee radiographs (anteroposterior and lateral view) were collected pre‐ and postoperatively, using the same PACS system (Agfa Impax 6.0) for every patient (Figures 1 and 2). Long‐leg images were required to show complete visualization of the hip, knee and ankle without cut‐off, with proper limb positioning and minimal rotation (assessed by symmetric appearance of the femoral condyles and the fibular overlap). Radiographs with poor exposure, rotational artifacts or incomplete visualization of the mechanical axis were excluded to ensure accuracy of the angular measurements. The following angles were measured:

- Hip–knee–ankle angle (HKA).
- Mechanical medial proximal tibial angle (MPTA): Values < 85° were considered suggestive of tibial‐based varus deformity [27].
- Mechanical lateral distal femoral angle (LDFA): Values > 90° were considered suggestive of a femoral‐based varus deformity [27].
- Joint line obliquity angle (JLO): This angle was considered in the safe zone if ≤ 4 [31].
- Joint line congruency angle (JLCA): If the angle opens laterally, it is considered suggestive of an intra‐articular deformity.
- Tibial bone varus angle (TBVA)—Levigne angle [20].

All radiological measurements were assessed twice, at least one month apart, by two independent orthopaedic surgeons (G. F. and M. D. P.). Intra‐rater and inter‐rater reliability were evaluated using intraclass correlation coefficients (ICCs) based on a two‐way mixed‐effects, absolute‐agreement model. According to Koo and Li's classification [19], ICC values < 0.5 indicate poor reliability, 0.5–0.75 moderate reliability, 0.75–0.90 good reliability and ≥0.90 excellent reliability.

Both intra‐rater and inter‐rater ICCs for all radiographic parameters demonstrated good to excellent reliability, confirming the consistency and reproducibility of the measurements.

### Statistical analysis

Statistical analysis was performed using R statistical software (Version 4.0.0; R Foundation for Statistical Computing) and GraphPad Prism Version 6.0 software (GraphPad Software Inc). The Shapiro–Wilk normality test was used to evaluate the normal distribution of the sample. Continuous variables are expressed as mean ± standard deviation and median [first quartile, third quartile]. Categorical variables are expressed in numbers of cases and frequencies; their differences were tested using the *χ* ^2^ test or Fisher's exact test. The between‐group differences for continuous variables were evaluated with the unpaired Student's *t* test or Mann–Whitney test, while the within‐group differences from baseline to final follow‐up for continuous variables were evaluated with the paired *t* test or Wilcoxon matched pairs signed‐rank test, according to the characteristics of the data distribution. For all analyses, the significance level was set at a *p* value < 0.05. Effect sizes (Cohen's *d*) were calculated only for between‐group comparisons at the last follow‐up using pooled standard deviations. No within‐group effect sizes were computed.

## RESULTS

**Figure 3:**
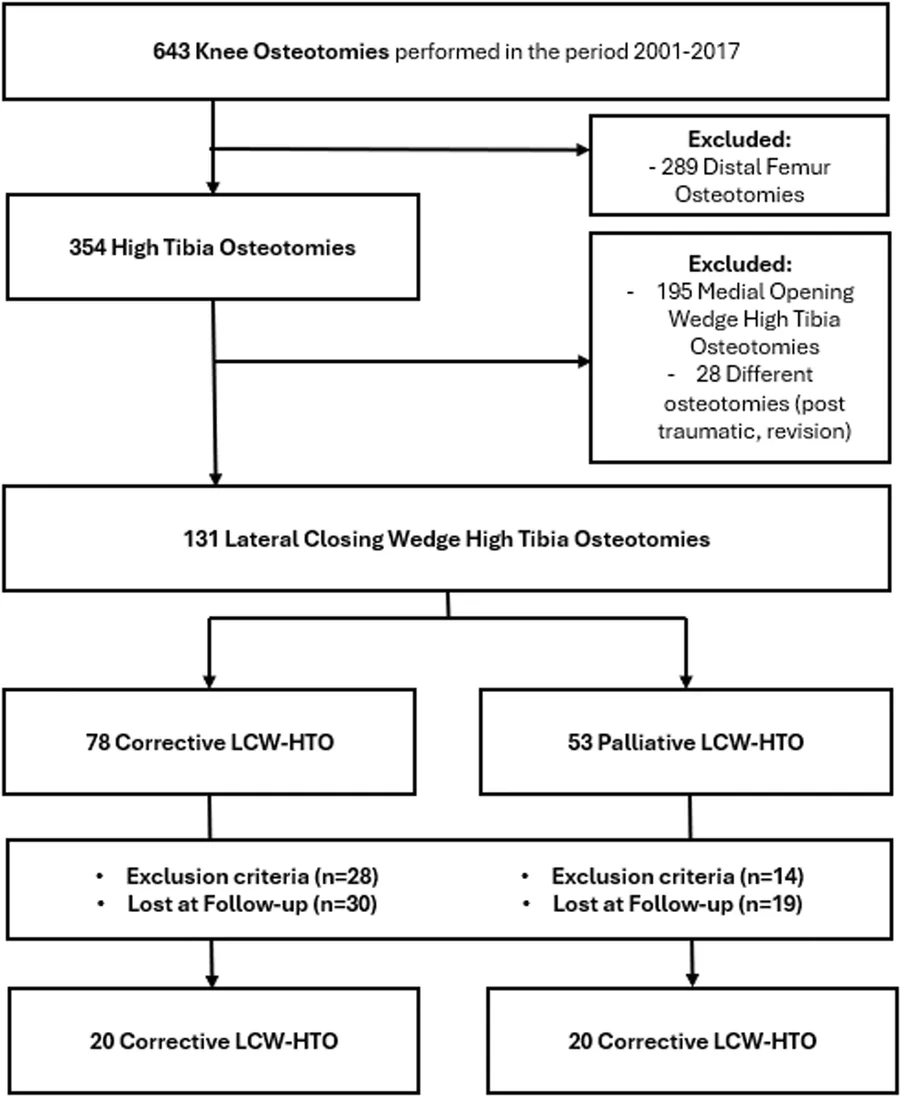
Flowchart of patients' selection. LCW‐HTO, lateral closing wedge‐high tibial osteotomy.

Out of a total of 643 knee osteotomies performed, 354 were HTOs. Among these, 131 were LCW‐HTOs. From this initial cohort of 131 patients (131 knees) with lower limb malalignment treated with LCW‐HTO, 40 patients (40 knees) met the inclusion criteria and were ultimately included in the study. The mean age at surgery was 49 ± 11 years (range, 45–57). Both PO and CO groups included 20 patients (20 knees), homogeneous by gender, body mass index (BMI) and age. Flow charts of patient selection and the study population demographics are available in Figure 3 and Table 1. No statistically significant differences were found between groups for any demographic variable (all *p* > 0.05).

**Table 1:** Demographics.

	Overall	CO group	PO group	*p* value
No. of patients	40	20	20	
No. of knees	40	20	20	
Follow‐up, y				
Mean	10.5 ± 2.9	11 ± 2.8	10.1 ± 3.0	0.34
Median	11.0 [5–16]	11.5 [9–16]	10.5 [5–13]	
Age at surgery, y				
Mean	49.2 ± 11.0	51.6 ± 10.6	46.8 ± 12.0	0.19
Median	53.0 [43–57]	55.5 [47–57]	51 [43–55]	
Gender				
Male	23 (57.5%)	14 (70%)	9 (45%)	0.20
Female	17 (42.5%)	6 (30%)	11 (55%)	
BMI at surgery (kg/m^2^)				
Mean	26.8 ± 2.6	27.0 ± 2.5	26.7 ± 2.7	0.72
Median	27.0 [25–28]	27.0 [25.7−28]	25.5 [24.7–29.0]	
Smoking				
Yes	16 (40%)	6 (30%)	10 (50%)	0.33
No	24 (60%)	14 (70%)	10 (50%)	

*Note*: Data are expressed as mean ± SD, median [Q1–Q3] or number of patients (percentage).

Abbreviations: BMI, body mass index; CO group, group of patients who received corrective osteotomy; No., number; PO group, group of patients who received palliative osteotomy; Q1, first quartile; Q3, third quartile; SD, standard deviation; y, years.

The mean overall HKA angle in the PO group before surgery was 175° ± 3.3° (range, 167°−178°) and in the CO group was 173.1° ± 3.0° (range, 167.5°−177.5°). Preoperative JLO did not differ between the PO group and the CO group (*p* > 0.05). Most postoperative values of JLO were within the safe zone of ≤4° in both groups: the postoperative JLO was >4° in five patients of the PO group (25%) and in two patients of the CO group (10%). Fisher's exact test for outliers was performed, resulting in PO knees exhibiting significantly greater postoperative JLO (mean postoperative JLO 3.5° ± 1.6°, range, 3°−6°) compared with CO knees (mean postoperative JLO 2.9° ± 1.5°, range, 2°−5°) (*p* < 0.05). The difference between the preoperative JLCA values in the two groups was not found to be significant as well (*p* > 0.05). The CO group presented a smaller JLCA postoperatively compared to the PO group (*p* < 0.05), and its value remained within the physiological limit (0°–2°), unlike the PO group, where its value exceeded the limit (mean value JLCA 3.2° ± 1.5°, range, 2°−6°). The postoperative MPTA in the CO group was 90° ± 3° (range, 85°–93°), while in the PO group it was 91° ± 3° (range, 88°–95°).

KL grading was evaluated preoperatively and at the last follow‐up in both groups. Preoperatively, 45% of patients were Grade 2, with similar distributions between groups (*p* > 0.05). Postoperatively, Grade 3 resulted as the most frequent (27.5%) (*p* > 0.05). Overall, 65% of patients showed no change in KL grade. Improvement by 1 or 2 grades occurred in 27.5% and 5.0% of patients, respectively. Additionally, the restoration of a neutral mechanical axis was achieved in both groups (overall mean HKA 179.4° ± 3°, range, 177°−186°). The preoperative Levigne angle showed a significant difference in both PO and CO groups (*p* < 0.05), even if the postoperative value was comparable without significant difference (*p* > 0.05).

As shown in Table 2, significant improvements in all clinical scores compared with baseline were observed in both groups (*p* < 0.01), with the only difference in favour of either group being a statistically significant higher value in preoperative and postoperative HSS for the PO group (*p* < 0.05). In the subgroup of patients with a postoperative JLO > 4°, clinical results showed significant improvement in all scores (*p* < 0.05) for both groups. The IKDC score increased from 52.9 ± 12.9 to 78.1 ± 10 in the CO group, and from 53.9 ± 11.8 to 81 ± 13.5 in the PO group (MCID = 12). The HSS objective and functional scores rose from 55 ± 11.8 to 79.9  ± 10.7 for CO, and from 63.4 ± 11.2 to 85.7 ± 12.9 for PO (MCID = 9). The Tegner activity scale improved from 1.5 ± 0.6 to 3.3 ± 1 for CO, and from 1.5  ±  0.6 to 2.8 ± 1.2 for PO (*p* < 0.05). The NRS score decreased from 77 ± 16.5 to 23.5 ± 14.2 for CO, and from 83 ± 17.2 to 22.5 ± 20.4 for PO (*p* < 0.05). Finally, the overall satisfaction rate was good as well in both PO and CO subgroups of patients, and all clinical improvements exceeded the established MCID thresholds: HSS (+≥9 points), IKDC (+≥12 points), Tegner (+0.5–1.0 levels) and NRS (−≥2 points or ≥30% reduction). Therefore, the magnitude of improvement observed in both groups was not only statistically significant but also clinically meaningful.

**Table 2:** Comparison of the radiological measurements and clinical outcomes, at baseline and last follow‐up, between the two groups, CO group versus PO group.

		CO group	PO group	*p* value	Cohen's *d*
HKA, deg	Pre‐Op	173.1 ± 3	175 ± 3.3	0.07	—
	Last FU	180.5 ± 2.3	178.9 ± 3.6	0.25	—
MPTA, deg	Pre‐Op	82.1 ± 1.7	86.3 ± 1.5	**<0.01**	—
	Last FU	90 ± 3	91 ± 3	0.37	—
mLDFA, deg	Pre‐Op	88.8 ± 2.7	88 ± 1.2	0.22	—
	Last FU	88.8 ± 2.7	88 ± 1.2	0.22	—
JLO, deg	Pre‐Op	2.7 ± 3	3.9 ± 2.5	0.31	
	Last FU	2.9 ± 1.5	3.5 ± 1.6	**0.02**	**−0.39**
JLCA, deg	Pre‐op	2.4 ± 2.2	3.5 ± 2.4	0.11	
	Last FU	1.6 ± 2.2	3.2 ± 1.5	**0.02**	**−0.83**
LEVIGNE, deg	Pre‐op	6.4 ± 2.7	3.1 ± 2.2	**<0.01**	—
	Last FU	2 ± 2.3	0 ± 3.5	**<0.01**	—
OA	Pre‐op	2.2	2	0.48	—
	Last FU	3.1	3.2	0.42	—
HSS	Pre‐op	55 ± 11.8	63.4 ± 11.2	**0.03**	
	Last FU	79.9 ± 10.7	85.7 ± 12.9	**0.04**	**−0.49**
IKDC	Pre‐op	52.9 ± 12.9	53.9 ± 11.8	0.81	
	Last FU	78.1 ± 10	81 ± 13.5	0.18	**−0.24**
TEGNER	Pre‐op	1.5 ± 0.6	1.5 ± 0.6	0.77	
	Last FU	3.3 ± 1	2.8 ± 1.2	0.14	**0.45**
NRS	Pre‐Op	77 ± 16.5	83 ± 17.2	0.10	
	Last FU	23.5 ± 14.2	22.5 ± 20.4	0.42	**0.06**
INSALL‐CROSBY	Last FU	Good	Good		

*Note*: Data are expressed as mean ± SD; statistically significant difference was achieved with a *p* value ≤ 0.05, represented in bold. Effect sizes shown in Table 2 refer exclusively to between‐group comparisons at the final follow‐up.

Abbreviations: CO, corrective osteotomy; FU, last follow‐up; HKA, hip–knee–ankle angle; HSS, Hospital for Special Surgery knee score; IKDC, subjective IKDC score; JLCA, joint line congruency angle; Insall–Crosby, Insall and Crosby grading system for patient's satisfaction; JLO, joint line obliquity angle; LEVIGNE, metaphyseal proximal tibial angle; mLDFA, mechanical lateral distal femoral angle; MPTA, medial proximal tibial angle; NRS, Numeric Rating Scale for pain; OA, osteoarthritis measured by Kellgren–Lawrence grading system; PO, palliative osteotomy; Pre‐op, preoperative/baseline; SD, standard deviation; Tegner, Tegner activity level scale.

Effect sizes (Cohen's *d*) and 95% confidence intervals were calculated for the main between‐group comparisons. The differences were small for HSS (*d* = −0.49, 95% confidence interval [CI]: −1.12 to 0.14) and IKDC (*d* = −0.24, 95% CI: −0.87 to 0.38), and negligible for Tegner (*d* = 0.45, 95% CI: −0.18 to 1.08) and NRS (*d* = 0.06, 95% CI: −0.56 to 0.68). Radiographic parameters showed a moderate effect size for JLCA (*d* = −0.83, 95% CI: −1.47 to −0.20) and a small effect for JLO (*d* = −0.39, 95% CI: −1.01 to 0.24), suggesting only limited clinical differences between groups.

Complete data about the clinical and radiological outcomes are reported in Table 2.

Arthroscopic treatment of associated lesions was performed in one patient (5%) in the CO group (medial condylar microfractures). In the PO group, six patients (30%) had undergone previous knee surgery, including four medial meniscectomies and two anterior cruciate ligament reconstructions. Considering intraoperative complications, one hinge fracture occurred in the CO group (Takeuchi Type I) (5%) [32], who then experienced hypertrophic nonunion and underwent a revision 1 year after the osteotomy (hardware removal, gap filling with synthetic bone graft and new fixation with plate and screws). In the PO group, one patient (5%) developed hardware intolerance, which was removed 6 months after the osteotomy, after complete healing. During the whole follow‐up period, three conversions to total knee arthroplasty (TKA) were recorded in the PO group (15%) and two in the CO group (10%) after a mean follow‐up of 11.2 ± 3.4 years (range, 7–14 years).

## DISCUSSION

The main finding of this study was that PO for varus knees in young patients produced clinical and radiographic outcomes comparable to those of CO, with no significant long‐term differences observed. Notably, JLO remained within acceptable limits (≤4°) in most cases, confirming the safety of PO in appropriately selected patients [31].

The so‐called ‘grey zone’ patients, those presenting with symptomatic varus alignment and intra‐articular pathology but without a correctable extra‐articular deformity, pose a particular treatment challenge, as they are often considered too young or active for arthroplasty. In such cases, PO serves as a joint‐preserving alternative that provides symptomatic relief while delaying the need for prosthetic replacement.

Knee osteotomy preserves the native joint and avoids the biomechanical drawbacks associated with prosthetic replacement. Jacquet et al. [15] reported a significant improvement in quality of life after osteotomy compared to UKA, particularly during the early postoperative period. However, compared to UKA, osteotomy is associated with a more demanding rehabilitation phase [17], requiring partial weight‐bearing and often causing discomfort due to fixation hardware. Despite these challenges, recent evidence demonstrates that knee osteotomy substantially improves quality of life and effectively postpones prosthetic replacement [21].

According to the recent European Society of Sports Traumatology, Knee Surgery and Arthroscopy (ESSKA) consensus recommendations [6, 7], optimal outcomes following knee osteotomy depend on performing the correction at the anatomical site of the deformity, when present. This strategy minimizes the risk of creating a compensatory deformity and ensures that the primary pathology is properly addressed, thereby improving both clinical and radiological results. Conversely, if the deformity is purely intra‐articular, osteotomy is generally considered an inappropriate indication.

From a biomechanical perspective, the main advantage of osteotomy lies in its ability to correct lower limb alignment and redistribute load across the knee, thereby unloading the affected compartment [13, 17]. Schröter et al. [30] demonstrated that even small alterations in the correction angle can markedly influence internal knee load distribution and enhance dynamic stability. This principle supports the rationale for PO, which typically involves modest angular corrections yet still yields meaningful clinical improvements [26].

Gaasbeek et al. [10] found that 75% of patients who underwent tibial valgus osteotomy did not require prosthetic replacement within 10 years, underscoring the procedure's effectiveness in delaying joint replacement.

In the present study, two patient groups were evaluated: the PO group, in which a PO was performed to correct a mild axial deformity primarily associated with intra‐articular pathology, and the CO group, in which the same surgical technique was applied to patients presenting with a more pronounced extra‐articular mechanical axis deformity.

The comparison revealed that, starting with a similar degree of preoperative HKA (173.1° ± 3° in CO vs. 175° ± 3.3° in PO) but at different degree of preoperative MPTA (82.1° ± 1.7° in CO vs. 86.3° ± 1.5° in PO), the only significant difference in terms of clinical outcomes was a higher HSS score for the PO group, while no difference was recorded in terms of OA progression.

Another significant difference observed was in postoperative JLO and JLCA, which were higher in the PO group than in the CO group. However, both remained within acceptable tolerance boundaries as described by other authors [25, 34].

There is still conflicting evidence regarding the association between postoperative JLO and clinical outcomes, as well as the acceptable tolerance limit for this parameter. The most recent study on JLO by Song et al. proposed 4 as an acceptable limit, noting clinical and radiographic worsening in patients with JLO exceeding 6 [31]. It is crucial to remember that static weight‐bearing radiographs do not entirely represent dynamic load distribution in the knee, as various studies have reported [29, 35, 36].

In the long term, a JLO that exceeds acceptable limits may predispose patients to premature osteotomy failure [23, 34], potentially requiring conversion to TKA or UKA. In the present study, with a mean follow‐up duration of 10.5 ± 2.9 years, three conversions to TKA (15%) were recorded in the PO group and two (10%) in the CO group. Importantly, all five conversions occurred more than ten years after the initial osteotomy, suggesting that excessive JLO was unlikely to be the main cause of failure. Moreover, although it is well‐established that osteotomy can complicate subsequent joint replacement procedures [16], no intraoperative complications or revision TKAs were reported among the prosthetic cases in our cohort.

The significantly lower postoperative JLCA observed in the CO group likely reflects a lower degree of intra‐articular deformity in these patients. This difference may help explain the greater variability in JLO noted within the PO group, where intra‐articular pathology had a more substantial influence on axial alignment. Collectively, these findings indicate that JLCA could serve as a valuable supplementary parameter when assessing the potential impact of osteotomy on postoperative joint line orientation.

### Limitations

This study presents certain limitations that should be acknowledged. First, its retrospective design introduces potential biases due to the lack of randomization. No priori power analysis was performed, and the relatively small sample size (20 patients per group) may limit the statistical power to formally demonstrate equivalence. However, the inclusion of all eligible cases treated with a uniform surgical technique and standardized follow‐up over a 16‐year period ensures a homogeneous cohort and supports the robustness and clinical relevance of the results. Although the study period (2001–2017) spans several years and a potential risk of heterogeneity cannot be entirely excluded, all procedures were performed by the same senior surgeon using a consistent lateral closing wedge technique and fixation method. Moreover, the postoperative rehabilitation protocol has remained substantially unchanged at our institution, minimizing the potential variability related to surgical technique or recovery management over time. The lack of biomechanical or gait analysis prevents a deeper understanding of the dynamic effects of JLO and alignment correction. Finally, while radiographic evaluations were consistent, the study did not account for potential confounding factors such as patient activity level or compliance with rehabilitation.

## CONCLUSIONS

PO may represent a viable joint‐preserving option for pathological varus knees in younger middle‐aged patients with mild intra‐articular deformity and medial unicompartmental knee OA. Despite achieving less optimal correction in JLO and JLCA compared to CO, the PO group showed similar trends in clinical scores, OA progression and conversion rates to knee arthroplasty. Larger cohort studies are warranted to confirm the role of PO.

## AUTHOR CONTRIBUTIONS

*Conception of the study*: Alessio Maione, Giuseppe Fedele and Pierrenzo Pozzi. *Acquisition of data*: Pierrenzo Pozzi, Giuseppe Fedele and Matteo Davide Parmigiani. *Analysis of data*: Pierrenzo Pozzi, Matteo Davide Parmigiani and Alessandra Menon. *Drafting of the work*: Pierrenzo Pozzi, Paolo Ferrua, Giuseppe Fedele, Alessio Maione, Filippo Calanna and Riccardo Compagnoni. *Revising it clinically for important intellectual content*: Paolo Ferrua, Filippo Calanna, Riccardo Compagnoni, Pietro Simone Randelli and Massimo Berruto. *Final approval of the version to be published*: Pierrenzo Pozzi, Alessio Maione, Paolo Ferrua, Riccardo Compagnoni, Pietro Simone Randelli and Massimo Berruto.

## CONFLICT OF INTEREST STATEMENT

The authors declare no conflicts of interest.

## ETHICS STATEMENT

The study was designed based on the criteria of the Declaration of Helsinki and approved by the local ethical committee (Comitato Etico Milano AREA 2—protocol number IOGPMB02).

## Data Availability

The data that support the findings of this study are available on request from the corresponding author. The data are not publicly available due to privacy or ethical restrictions.

## References

[1] Aygün Ü , Bölükbaşı M , Yamak K , Çiçek AC . Comparison of the Miniaci and Dugdale techniques on functional outcomes in medial open wedge high tibial osteotomy. J Exp Orthop. 2023;10(1):53. 10.1186/s40634-023-00653-5 PMC10449725 37615790

[2] Bilgasem A , Vivekanantha P , Gyemi L , Hassan Z, Slawska‐Eng D, Meena A, et al. Large variability in MCID, SCB and PASS values among literature investigating patellar stabilization surgery: a systematic review. Knee Surg Sports Traumatol Arthrosc. 2025. 10.1002/ksa.12684 PMC12850594 40326338

[3] Bonasia DE , Dettoni F , Sito G , Blonna D , Marmotti A , Bruzzone M , et al. Medial opening‐wedge high tibial osteotomy for medial compartment overload/arthritis in the varus knee: prognostic factors. Am J Sports Med. 2014;42(3):690–698. 10.1177/0363546513516577 24449807

[4] Constantin H , Salmon LJ , Russell V , Sundaraj K , Roe JP , Pinczewski LA . 20‐year outcomes of high tibial osteotomy: determinants of survival and functional outcome. Am J Sports Med. 2024;52(2):344–351. 10.1177/03635465231217742 38243788

[5] Crosby E , Insall J . Recurrent dislocation of the patella: relation of treatment to osteoarthritis. The Journal of Bone & Joint Surgery. 1976;58:9–13. 1249117

[6] Dawson M , Elson D , Claes S , Predescu V , Khakha R , Espejo‐Reina A , et al. Osteotomy around the painful degenerative varus knee has broader indications than conventionally described but must follow a strict planning process: ESSKA formal consensus part I. Knee Surg Sports Traumatol Arthrosc. 2024;32(7):1891–1901. 10.1002/ksa.12256 38738832

[7] Dawson MJ , Ollivier M , Menetrey J , Beaufils P . Osteotomy around the painful degenerative varus knee: a 2022 ESSKA formal consensus. Knee Surg Sports Traumatol Arthrosc. 2023;31(8):3041–3043. 10.1007/s00167-022-07024-0 35697873

[8] Fan XY , Ma JH , Wu X , Xu X , Shi L , Li T , et al. How much improvement can satisfy patients? Exploring patients' satisfaction 3 years after total knee arthroplasty. J Orthop Surg. 2021;16(1):389. 10.1186/s13018-021-02514-2 PMC8212506 34140037

[9] Fitoussi A , Dartus J , Erivan R , Pasquier G , Migaud H , Putman S , et al. Management of medial femorotibial osteoarthritis: epidemiology and survival of unicompartmental knee arthroplasty versus valgus high tibial osteotomy in France: study of 108,007 cases from the French National Hospitals Database. Orthop Traumatol Surg Res. 2023;109(8):103692. 10.1016/j.otsr.2023.103692 37776952

[10] Gaasbeek RDA , Nicolaas L , Rijnberg WJ , Van Loon CJM , Van Kampen A . Correction accuracy and collateral laxity in open versus closed wedge high tibial osteotomy. A one‐year randomised controlled study. Int Orthop. 2010;34(2):201–207. 10.1007/s00264-009-0861-7 PMC2899362 19707760

[11] Hawker GA , Mian S , Kendzerska T , French M . Measures of adult pain: VAS, NRS, MPQ, SF‐MPQ, CPGS, SF‐36 BPS, and ICOAP. Arthritis Care Res (Hoboken). 2011;63(Suppl 11):S240–S252. 10.1002/acr.20543 22588748

[12] Hirschmann MT , Moser LB , Amsler F , Behrend H , Leclercq V , Hess S . Phenotyping the knee in young non‐osteoarthritic knees shows a wide distribution of femoral and tibial coronal alignment. Knee Surg Sports Traumatol Arthrosc. 2019;27(5):1385–1393. 10.1007/s00167-019-05508-0 30980119

[13] Hohloch L , Kim S , Mehl J , Zwingmann J , Feucht MJ , Eberbach H , et al. Customized post‐operative alignment improves clinical outcome following medial open‐wedge osteotomy. Knee Surg Sports Traumatol Arthrosc. 2018;26(9):2766–2773. 10.1007/s00167-017-4731-3 28975376

[14] Irrgang JJ , Anderson AF , Boland AL , Harner CD, Kurosaka M, Neyret P, et al. Development and validation of the International Knee Documentation Committee Subjective Knee Form. Am J Sports Med. 2001;29(5):600–613 10.1177/03635465010290051301 11573919

[15] Jacquet C , Gulagaci F , Schmidt A , Pendse A , Parratte S , Argenson JN , et al. Opening‐wedge high tibial osteotomy allows better outcomes than unicompartmental knee arthroplasty in patients expecting to return to impact sports. Knee Surg Sports Traumatol Arthrosc. 2020;28(12):3849–3857. 10.1007/s00167-020-05857-1 32008058

[16] Jin C , Song EK , Santoso A , Ingale PS , Choi IS , Seon JK . Survival and risk factor analysis of medial open‐wedge high tibial osteotomy for unicompartment knee osteoarthritis. Arthroscopy. 2020;36(2):535–543. 10.1016/j.arthro.2019.08.040 31901391

[17] Kayaalp ME , Apseloff NA , Lott A , Kaarre J , Hughes JD , Ollivier M , et al. Around‐the‐knee osteotomies part 1: definitions, rationale and planning—state of the art. J ISAKOS. 2024;9(4):645–657. 10.1016/j.jisako.2024.02.017 38460600

[18] Kellgren JH , Jeffrey M , Ball J . Atlas of standard radiographs of arthritis. Oxford: Blackwell Scientific Publications; 1963.

[19] Koo TK , Li MY . A guideline of selecting and reporting intraclass correlation coefficients for reliability research. J Chiropr Med. 2016;15(2):155–163. 10.1016/j.jcm.2016.02.012 PMC4913118 27330520

[20] Lévigne C , Dejour D , Neyret P . Intérêt de l′axe épiphysaire dans l′arthrose. Journées Lyon Chir Genou. 1998;7:85–92.

[21] Lott A , James MG , Kaarre J , Höger S , Kayaalp ME , Ollivier M , et al. Around‐the‐knee osteotomies part II: surgical indications, techniques and outcomes—state of the art. J ISAKOS. 2024;9(4):658–671. 10.1016/j.jisako.2024.04.002 38604568

[22] Micicoi G , Khakha R , Kley K , Wilson A , Cerciello S , Ollivier M . Managing intra‐articular deformity in high tibial osteotomy: a narrative review. J Exp Orthop. 2020;7(1):59. 10.1186/s40634-020-00283-1 PMC7481321 32902758

[23] Na YG , Lee BK , Choi JU , Lee BH , Sim JA . Change of joint‐line convergence angle should be considered for accurate alignment correction in high tibial osteotomy. Knee Surg Relat Res. 2021;33(1):4. 10.1186/s43019-020-00076-x PMC7798206 33431062

[24] Ollivier B , Berger P , Depuydt C , Vandenneucker H . Good long‐term survival and patient‐reported outcomes after high tibial osteotomy for medial compartment osteoarthritis. Knee Surg Sports Traumatol Arthrosc. 2021;29(11):3569–3584. 10.1007/s00167-020-06262-4 32909057

[25] Ollivier M , An JS , Kley K , Khakha R , Fernandes LR , Micicoi G . A significant rate of tibial overcorrection with an increased JLO occurred after isolated high tibial osteotomy without considering international consensus. Knee Surg Sports Traumatol Arthrosc. 2023;31(11):4927–4934. 10.1007/s00167-023-07518-5 37597039

[26] Ollivier M , Claes S , Mabrouk A , Elson D , Espejo‐Reina A , Predescu V , et al. Surgical strategy and complication management of osteotomy around the painful degenerative varus knee: ESSKA formal consensus part II. Knee Surg Sports Traumatol Arthrosc. 2024;32(8):2194–2205. 10.1002/ksa.12273 38769785

[27] Paley D . Normal lower limb alignment and joint orientation. In: Paley D , editor. Principles of deformity correction. Berlin, Heidelberg: Springer; 2022. p. 1–18.

[28] Palmer J , Getgood A , Lobenhoffer P , Nakamura R , Monk P . Medial opening‐wedge high tibial osteotomy for the treatment of medial unicompartmental knee osteoarthritis: a state‐of‐the‐art review. J ISAKOS. 2024;9(1):39–52. 10.1016/j.jisako.2023.10.004 37839705

[29] Razak HRBA , Micicoi G , Khakha RS , Ehlinger M , Faizan A , LiArno S , et al. Patients with varus knee osteoarthritis undergoing high tibial osteotomy exhibit more femoral varus but similar tibial morphology compared to non‐arthritic varus knees. Knee Surg Sports Traumatol Arthrosc. 2022;30(2):680–687. 10.1007/s00167-020-06426-2 33423093

[30] Schröter S , Nakayama H , Ihle C , Ahrend MD , Kuwashima U . Minimal‐invasive biplanare closed‐wedge‐DFO (distale femurosteotomie). Knie J. 2020;2(3):212–219.

[31] Song JH , Bin SI , Kim JM , Lee BS . What is an acceptable limit of joint‐line obliquity after medial open‐wedge high tibial osteotomy? Analysis based on midterm results. Am J Sports Med. 2020;48(12):3028–3035. 10.1177/0363546520949552 32941061

[32] Takeuchi R , Ishikawa H , Kumagai K , Yamaguchi Y , Chiba N , Akamatsu Y , et al. Fractures around the lateral cortical hinge after a medial opening‐wedge high tibial osteotomy: a new classification of lateral hinge fracture. Arthroscopy. 2012;28(1):85–94. 10.1016/j.arthro.2011.06.034 21982387

[33] Tegner Y , Lysholm J . Rating systems in the evaluation of knee ligament injuries. Clin Orthop Relat Res. 1985;198:42–49. 4028566

[34] Xie T , Brouwer RW , van den Akker‐Scheek I , van der Veen HC . Clinical relevance of joint‐line obliquity after high tibial osteotomy for medial knee osteoarthritis remains controversial: a systematic review. Knee Surg Sports Traumatol Arthrosc. 2023;31(10):4355–4367. 10.1007/s00167-023-07486-w PMC10471655 37340220

[35] Xie T , de Vries AJ , van der Veen HC , Brouwer RW . Influence of increased joint‐line obliquity on survivorship after lateral closing‐wedge high tibial osteotomy. Am J Sports Med. 2024;52:2792–2798. 10.1177/03635465241270292 PMC11408944 39165165

[36] Xie T , Huizinga MR , van den Akker‐Scheek I , van der Veen HC , Brouwer RW . Joint‐line obliquity after lateral closing‐wedge high tibial osteotomy does not adversely affect clinical and radiological outcome: a 5‐year follow‐up study. Knee Surg Sports Traumatol Arthrosc. 2023;31(11):4851–4860. 10.1007/s00167-023-07532-7 PMC10598188 37561185

